# Relationship between change in body composition and cardiorespiratory fitness during cardiac rehabilitation in patients with cardiovascular disease

**DOI:** 10.3389/fendo.2026.1933372

**Published:** 2026-09-22

**Authors:** Salaheddin M. Sharif, Jenna L. Taylor, Amanda R. Bonikowske, Thomas P. Olson

**Affiliations:** 1 Department of Cardiovascular Medicine, Mayo Clinic, Rochester, MN, United States; 2 Montana College of Osteopathic Medicine, Rocky Vista University, Billings, MT, United States; 3 School of Human Movement and Nutrition Sciences, The University of Queensland, Brisbane, QLD, Australia

**Keywords:** CPET, DEXA, exercise training, non-responders, responders, VO_2_peak

## Abstract

**Background:**

Cardiac rehabilitation (CR) improves peak oxygen uptake (VO_2_peak) and body composition in patients with cardiovascular disease. However, improvement in VO_2_peak is not observed in all patients following a CR program, and whether VO_2_peak non-responders also demonstrate limited changes in body composition remains unclear. This study aimed to examine the association between changes in VO_2_peak and body composition in patients undergoing a CR program.

**Methods:**

Retrospective analysis of patients who completed CR between 2015 and 2022 at Mayo Clinic. The study included 143 patients who completed cardiopulmonary exercise testing and dual-energy X-ray absorptiometry pre- and post-CR. Patients were grouped by the change in relative VO_2_peak response to CR (responders= Δ>0 vs non-responders=Δ ≤0 mL/kg/min). Two-way repeated ANOVA was used to compare groups, and regression models were used to examine the associations between baseline body composition and changes in body composition with VO_2_peak change.

**Results:**

Among the cohort, 26% were classified as non-responders based on relative VO_2_peak measurements. Both groups exhibited a significant improvement in most body composition variables including body fat mass, body fat percentage, body lean percentage, and fat mass index (all p<0.001), with no differences in the percentage of change in body composition variables between groups (p>0.05). Regression models demonstrated that neither baseline body composition nor changes in body composition were significantly associated with changes in VO_2_peak.

**Conclusion:**

Our data suggest that baseline and change in body composition were not significantly associated with changes in VO_2_peak in patients undergoing the CR program.

## Introduction

Cardiorespiratory fitness (CRF) is a powerful predictor of all-cause and cardiovascular disease (CVD) related mortality (1). Studies have indicated that every 1% increase in peak oxygen consumption (VO_2_peak) confers a 2% reduction in CVD‐related mortality over 5 years in coronary artery disease (CAD) patients (2). Baseline VO_2_peak and its improvement during cardiac rehabilitation (CR) strongly predict future prognosis, including the risk of CVD readmissions and all-cause mortality (3). Multiple studies have confirmed that CR program improves CVD risk factors, including CRF, psychological risk factors, quality of life (QoL), morbidity, CVD mortality, and all-cause mortality (4–6). Improvement in CRF appears to be a key factor contributing to improvement in psychological health and mortality benefits following CR program (7, 8). However, several studies have shown that VO_2_peak does not increase in all patients (non-responders) after CR (9–13). The percentage of non-responders among those who completed CR program ranges from 21% to 23% (10, 11). This is critically important as improvement in VO_2_peak is associated with a higher survival rate in those patients (3, 11, 14).

While the underlying mechanisms and factors related to the lack of VO_2_peak improvement have not been fully elucidated, several studies have identified common predictors of increased VO_2_peak after a CR program, including age, sex, body mass index (BMI), and baseline VO_2_peak (11, 15, 16). Recently, we reported that younger age, lower baseline VO_2_peak, and higher number of CR sessions are independent predictors of VO_2_peak improvement (10). Obesity-related impairments in CRF were observed, with greater adiposity corresponding to lower VO_2_peak (17). Interestingly, a recent study has demonstrated that CAD patients with higher body fat percentage (BF%) and lower lean body mass (LBM) have a greater rate of long‐term major adverse cardiovascular events (18), with LBM closely correlated with physical function performance and VO_2_peak (19, 20). Although the CR program is also well known to improve body composition (21–23), the interrelationship between the CR-related changes in VO_2_peak and improvement in body composition is not well understood among patients undergoing the CR program. Therefore, this study aimed to examine the relationship between change in body composition and change in VO_2_peak and determine whether baseline body composition was associated with VO_2_peak improvements in CR patients.

## Methods

### Study design

These retrospective data were previously reported in a study that addressed a different research question using a distinct analytical approach (24). This retrospective cohort study included consecutively enrolled patients who completed an outpatient CR program between January 1, 2015, and October 31, 2022, at Mayo Clinic, Rochester, MN. All patients were residents of Olmsted County, aged 18 years or older, and had a cardiac event or procedure that served as a clinical indication for the CR program. We included all patients during this timeframe who completed a cardiopulmonary exercise test (CPET) and completed dual-energy X-ray absorptiometry (DEXA) pre- and post-CR participation. Demographic data, clinical characteristics, indications for CR program, and the interval between CPETs were obtained from an institutional database. This study was approved by the Mayo Clinic Institutional Review Board (16-009071) and complied with the research authorization protocol for the use of medical records as required by the state of Minnesota. All included patients had authorized the use of their medical records for research purposes in accordance with Minnesota law.

### Cardiopulmonary exercise test

Patients completed pre- and post-CR assessments using an institutionally designed incremental exercise protocol on either a treadmill or cycle ergometer under cardiologist supervision, as previously described (21, 22). This protocol consisted of increasing 2 metabolic equivalents every 2 minutes with an endpoint of volitional fatigue. Heart rate (HR) and rhythm were monitored continuously with a 12-lead ECG, and blood pressure was measured at the end of each stage by manual sphygmomanometry. Ratings of perceived exertion (RPE: Borg 6–20 scale) were recorded at the end of each stage. Flow and gas exchange variables were measured breath-by-breath during exercise using indirect calorimetry (MGC Diagnostics). Measured variables included VO_2_, carbon dioxide production, tidal volume, and respiratory rate. The VO_2_, anaerobic threshold (AT), respiratory exchange ratio (RER), and peak oxygen (O_2_) pulse were calculated. To ensure that patients reach maximal effort during CPET, the criteria include an RER > 1.10, achieving ≥85% of age-predicted maximum HR, and a high Borg RPE score (≥17) or signs of volitional fatigue. Peak values were obtained by averaging the last 30 seconds of the test. Cardiac medications were not withheld before CPET to maximize patient safety and generalizability of the data. Heart rate recovery (HRR) was calculated as the difference between peak and 1-minute post-exercise HR. Given our aim, we pursued a relative VO_2_ that is used to differentiate between responders and non-responders. We calculated the degree of VO_2_peak improvement post-CR as a change in relative VO_2_peak (mL/kg/min). Consistent with the methodology previously described the cohort was classified into responders (ΔVO_2_peak>0 mL/kg/min) and non-responders (ΔVO_2_peak ≤ 0 mL/kg/min) (13). This classification was chosen to maintain methodological consistency with prior studies and to facilitate direct comparison of interindividual variability in the VO_2_peak response to the CR program. Because this zero-based threshold does not account for the technical measurement error or biological variability inherent to VO_2_peak assessment, these terms indicate the presence or absence of an observed increase in VO_2_peak rather than definitive physiological response phenotypes (25). As a complementary analysis, participants were also classified according to the change in absolute VO_2_peak (L/min) to assess whether the findings were consistent when the influence of body mass.

### Dual-energy X-ray absorptiometry

Body composition was assessed using dual-energy X-ray absorptiometry (DEXA; GE Lunar iDXA Series X, Madison, WI, USA) using standardized procedures as described previously (26). Baseline DEXA assessments were completed before enrollment in the CR program, whereas post-CR DEXA assessments were completed within 4 weeks after CR graduation. Scans were used to measure total body mass (BM), BLM, and body fat mass (BFM). Body mass index, body lean mass index (LMI), and body fat mass index (FMI) were calculated as mass divided by height squared. Body lean percentage (BL%) and BF% were calculated as BLM or BFM divided by BM multiplied by 100. The scanner was calibrated daily against a manufacturer-supplied standard calibration block to control for possible baseline drift.

### Cardiac rehabilitation

Our comprehensive CR program is based on the guidelines of the American Association of Cardiovascular and Pulmonary Rehabilitation, American Heart Association, and American College of Cardiology as described previously (16, 27). In our CR program, patients were prescribed 36 supervised sessions that generally occurred 2–3 days per week over 12–18 weeks (22). However, following the COVID-19 pandemic, adjustments were made to our CR program to ensure the safety and continuity of care. As a result, the number of supervised sessions was reduced 24, and the frequency was changed to twice a week to accommodate social distancing measures, patient concerns, and resource limitations while maintaining effective CR protocols. Exercise prescriptions were individualized and revised as the patients progressed through the program. Patients participated in 30–45 minutes of supervised aerobic activity and an additional 10–15 minutes of resistance training. The primary method for prescribing intensity during aerobic exercise was the Borg 6–20 RPE scale. Moderate-intensity training was performed for 20–45 min at an RPE of 12–14 in the first 2 weeks of the CR program. In the 3^rd^ week, patients were instructed to perform high-intensity interval training at an RPE of 15-18. The initial protocol consisted of four high-intensity intervals lasting 30–60 s at an RPE of 15–17, interspersed with 1–5 minutes of lower-intensity exercise at an RPE <14. Based on individual tolerance and clinical status, the protocol was progressively advanced to 5–8 high-intensity intervals lasting 2–4 minutes within a 20–45-minute aerobic exercise session, in accordance with American College of Sports Medicine guidelines (21, 22, 28). Patients were also urged to complete at least 30 minutes of moderate-intensity physical activity at home on days without supervised CR sessions. Additional education was delivered to CR patients, including healthy nutrition, stress management, medication management and adherence, cardiovascular risk factor control, and proper sleep hygiene.

### Statistical analyses

Patient baseline characteristics are presented as mean and standard deviation (SD) and categorical data were summarized as counts and percentages. Baseline characteristics of responders and non-responders were compared by independent t-tests for continuous data and Fisher’s exact test for categorical data. The pre-CR to post-CR change for each variable was analyzed within the whole cohort using a paired t-test. Normality was tested using the Shapiro–Wilk test. If the normality test failed, log transformation was used. Two-way repeated measures (group x time) analysis of variance (ANOVA) was used to determine the main group effect, the main time effect, and the group x time interaction effect. If a significant F value was observed, Tukey’s *post hoc* testing was performed. The percentage change (%Δ) from pre- to post-CR for each variable was calculated and %Δ between groups was analyzed using an unpaired t-test. Regression models were used to evaluate the associations of baseline body composition variables and changes in body composition with changes in VO_2_peak. Statistical significance was defined as a P value of <0.05. All statistical analysis were completed using SigmaPlot (Palo Alto, CA), and graphic presentation was completed using GraphPad Prism (San Diego, CA).

## Results

### Whole cohort

In the study cohort, 143 patients were included with a mean age of 63 ± 12 years and a BMI of 30 ± 6 kg/m^2^. Patients completed an average of 27 ± 11 supervised CR sessions, with CPET conducted predominantly on a treadmill (94%). The mean absolute VO_2_peak and relative VO_2_peak increased significantly from pre-CR to post-CR (absolute VO_2_peak: 2.04 ± 0.7 vs 2.14 ± 0.7 L/min; and relative VO_2_peak: 22.4 ± 6 vs 23.9 ± 6 mL/kg/min; all p<0.001). For absolute VO_2_peak, this was a mean improvement of 0.102 ± 0.3 L/min and a %Δ of 6 ± 13%. Additionally, for relative VO_2_peak, this was a mean improvement of 1.6 ± 3 mL/kg/min and Δ% of 8 ± 14%. Body composition analysis demonstrated a significant improvement in BM (91.1 ± 21 vs 89.9 ± 20 kg, p<0.001), LBM (55.3 ± 11 vs 55.7 ± 11 kg, p=0.003), BFM (33 ± 13 vs 31 ± 12 kg, p<0.001), BL% (61 ± 8 vs 63 ± 8%, p<0.001), and BF% (35 ± 8 vs 34 ± 8%, p<0.001). In addition, there was improvement in BMI (30.3 ± 6 vs 29.9 ± 6 kg/m^2^, p<0.001), LMI (18.3 ± 3 vs 18.5 ± 2 kg/m^2^, p=0.002), and FMI (10.9 ± 4 vs 10.3 ± 4 kg/m^2^, p<0.001).

### Relative VO_2_peak response with cardiac rehabilitation

The distribution of Δ% relative VO_2_peak for the cohort is shown in **Figure 1**. We found that 74% (n=106) of patients (responders) responded to exercise training and 26% (n=37) of patients (non-responders) did not respond to exercise training during the CR program. At baseline, there was no difference in the characteristics of patients, the indication for CR, duration between CPET, and CPET exercise modality between responders and non-responders (**Table 1**; p>0.05). However, non-responders had a lower number of CR sessions (**Table 1**; p<0.001).

**Figure 1:**
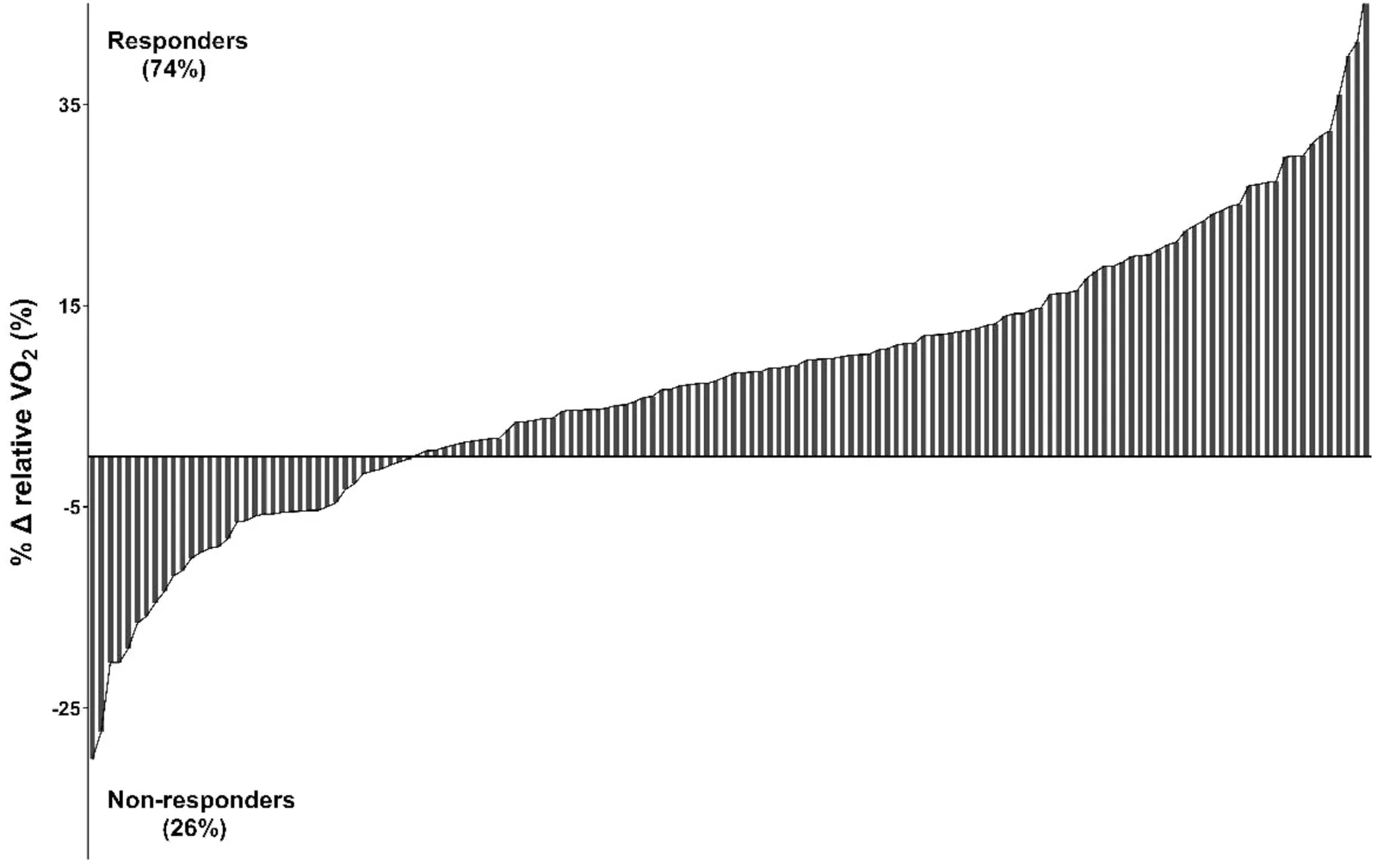
The distribution of percent change of peak oxygen consumption in the whole cohort.

**Table 1:** Baseline characteristics.

	Responders (n=106)	Non-responders (n=37)	P-value
Characteristic			
Age (years)	64 ± 13	61 ± 12	0.26
Female (%)	22 (21%)	6 (16%)	0.64
Body weight (kg)	90 ± 20	95 ± 22	0.19
Height (cm)	172 ± 9	175 ± 8	0.13
VO_2_ (mL/kg/min)	22.3 ± 6	23.0 ± 7	0.55
CR indication			
Angina	7 (7%)	5 (14%)	0.29
Acute MI	15 (14%)	10 (27%)	0.08
NSTEMI	11 (10%)	2 (5%)	0.99
STEMI	6 (6%)	1 (3%)	0.46
PCI	52 (49%)	15 (41%)	0.45
Surgery	13 (12%)	2 (5%)	0.35
Other	2 (2%)	2 (5%)	0.28
Duration between CPET (weeks)	15 ± 5	15 ± 3	0.84
CR sessions	29 ± 9	22 ± 12	<0.001
CPET modality TM (%)	100 (94%)	35 (95%)	0.99

Data presented as mean ± SD or number (%) as appropriate. CR, cardiac rehabilitation; MI, myocardial infarction; NSTEMI, non-ST-elevation myocardial infarction; STEMI, ST-elevation myocardial infarction; PCI, percutaneous coronary intervention; Surgery, coronary artery bypass graft, valves replacement; Other, heart failure, cardiac arrest; CPET, cardiopulmonary exercise test; TM, treadmill.

The CPET data of responders and non-responders are presented in **Table 2**. In responders, both absolute VO_2_peak and relative VO_2_peak increased significantly by 0.21 ± 0.2 L/min (2.0 ± 0.7 vs 2.21 ± 0.7 L/min) and 2.86 ± 2 mL/kg/min (22.3 ± 6 vs 25.1 ± 6 mL/kg/min) respectively (both p<0.001). For non-responders, both absolute VO_2_peak and relative VO_2_peak decreased significantly by 0.21 ± 0.2 L/min (2.12 ± 0.6 vs 1.92 ± 0.6 L/min) and 2.1 ± 2 mL/kg/min (22.9 ± 7 vs 20.9 ± 7 mL/kg/min), respectively (all p<0.001). In addition, the %Δ relative VO_2_peaks significantly differed between responders and non-responders (14 ± 10 vs -7 ± 9%; p<0.001). There was a significant time effect observed for rest HR and HRR (**Table 2**; p<0.05). *Post hoc* analysis revealed a decrease in both groups for rest HR and only an increase in HRR within responders (all p<0.05). For peak HR, a significant time and interaction effect was observed (**Table 2**; p<0.05), with *post hoc* analysis revealing an increase among responders, a decrease among non-responders, and a difference between groups at post-CR (all p<0.05). There was a significant interaction effect for AT and O_2_ pulse (**Table 2**; p<0.05), and *post hoc* analysis revealed an increase within responders only (p<0.05). For exercise time, significant time and interaction effects were observed (**Table 2**; p<0.001), with *post hoc* analysis revealing an increase among responders and a difference between groups at post-CR (both p<0.05). For systolic blood pressure (SBP), RER, and RPE, there were no significant differences between or within groups (p>0.05). Regarding %Δ between responders and non-responders, there was a significant difference in %Δ exercise time (16 ± 19 vs 2 ± 15%), %Δ peak HR (4 ± 11 vs -6 ± 12%), %Δ AT (8 ± 29 vs -7 ± 29%), and %Δ O_2_ pulse (7 ± 13 vs -3 ± 11%) (**Figure 2**; all p<0.05). There was no difference in %Δ rest HR (-4 ± 15 vs -6 ± 15%) and % ΔHRR (17 ± 230 vs -1 ± 149%) between groups (both p>0.05).

**Table 2:** Cardiopulmonary exercise test results between responders and non-responders.

CPET variable	Responders		Non-responders		P-value		
	Pre-CR	Post-CR	Pre-CR	Post-CR	Group	Time	Interaction
Rest HR (beat/min)	69 ± 13	66 ± 13*	73 ± 14	68 ± 13*	0.19	<0.001	0.43
Peak HR (beat/min)	134 ± 22	138 ± 19*#	139 ± 24	129 ± 24*	0.57	0.04	<0.001
HRR (beat/min)	16 ± 10	19 ± 10*	15 ± 10	16 ± 10	0.30	0.03	0.28
SBP (mm Hg)	167 ± 29	168 ± 26	172 ± 29	164 ± 30	0.90	0.15	0.09
RER	1.15 ± 0.1	1.16 ± 0.1	1.16 ± 0.1	1.15 ± 0.1	0.73	0.74	0.63
VO_2_ at AT (L/min)	1.48 ± 0.5	1.54 ± 0.5*	1.50 ± 0.4	1.38 ± 0.4	0.67	0.98	<0.01
O_2_ pulse (mL/beat)	15.0 ± 5	15.8 ± 5*	15.4 ± 3	15.3 ± 3	0.91	0.31	<0.001
RPE	18.2 ± 0.7	18.3 ± 0.7	18.1 ± 0.9	18.2 ± 0.5	0.07	0.62	0.86
Exercise time (min)	7.6 ± 2	8.7 ± 2*#	7.7 ± 2	7.8 ± 2	0.17	<0.001	<0.001

Data presented as mean ± SD. CR, cardiac rehabilitation; HR, heart rate; HRR, heart rate recovery; SBP, systolic blood pressure; RER, respiratory exchange ratio; AT, anaerobic threshold; O_2_ pulse, oxygen pulse; RPE, ratings of perceived exertion.

^*^ P<0.05, a significant difference between Pre and Post within the group.

^#^ P<0.05, a significant difference between groups within the Post-CR.

**Figure 2:**
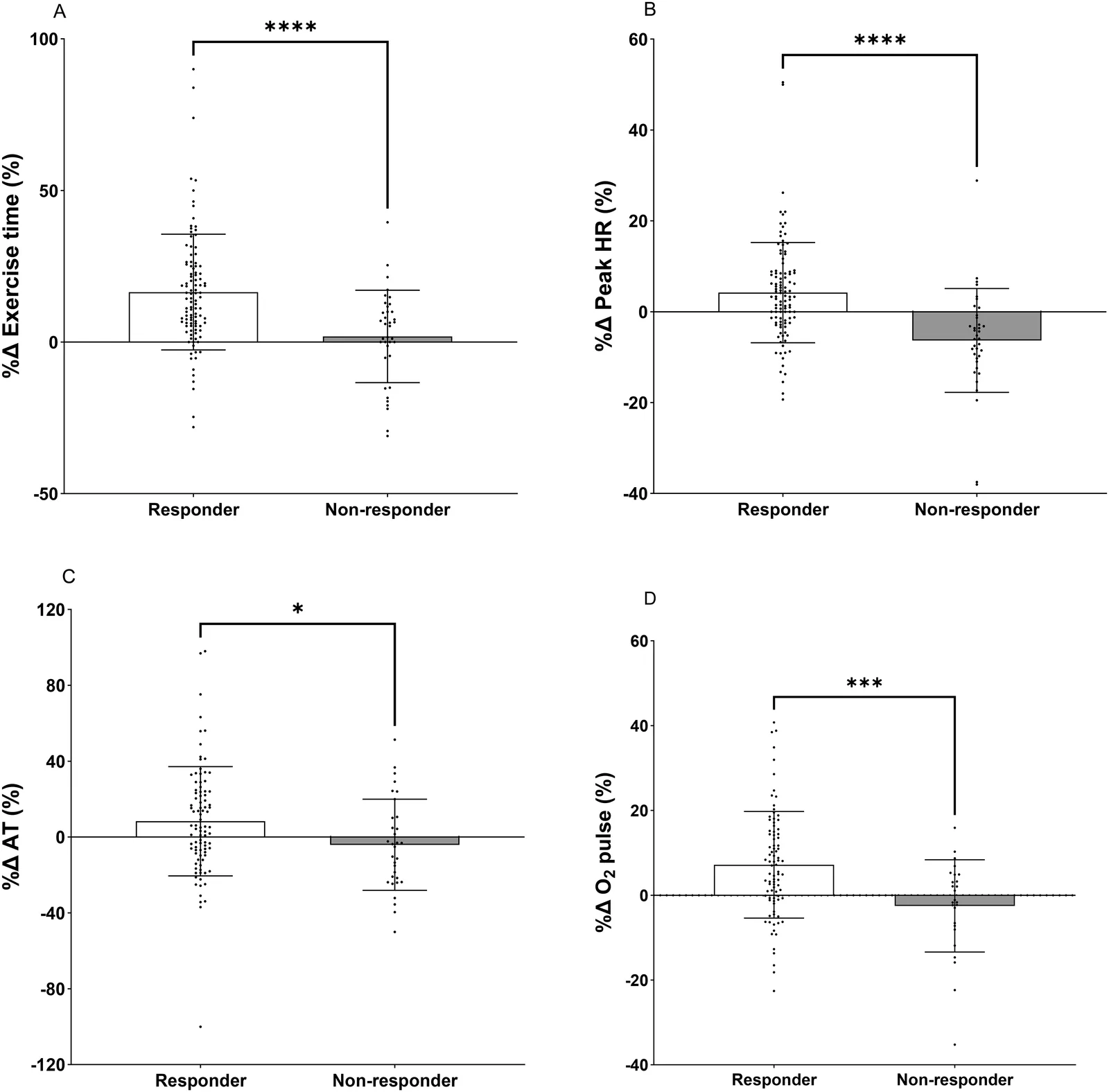
The percent change of cardiopulmonary exercise test variables between responders and non-responders in cohort according to peak relative oxygen consumption. **(A)** Percent change of exercise time **(B)** Percentage change of peak heart rate. **(C)** Percent change of anaerobic threshold. **(D)** Percent change of oxygen pulse. ****P<0.0001, ***P<0.01, *P<0.05.

No significant interaction effects were observed for any body composition variable (all p>0.05, **Table 3**). A significant main effect of time was observed for BM (**Table 3**; p<0.001), with *post hoc* analysis showing a decrease in responders (p<0.05). For LBM, there was a significant group and time effect (**Table 3**; p<0.05) with an increase in responders and the difference between groups at post-CR (all p<0.05). For BFM, BL%, and BF%, there was a significant time effect (**Table 3**; all p<0.001), and *post hoc* analysis revealed improvement in both groups (all p<0.05). In addition, there was a significant time effect (**Table 3**; p<0.01) observed for both BMI and LMI and *post hoc* analysis revealed a decrease in BMI and an increase in LMI in responders (p<0.05). For FMI, there was a significant time effect (**Table 3**; p<0.001) and *post hoc* analysis revealed a decrease in both groups (p<0.05). There was no significant difference between responders and non-responders in %Δ LBM (0.9 ± 3 vs 0.8 ± 3%), %Δ BFM (-5.3 ± 9 vs -3.7 ± 5%), %Δ BL% (2.4 ± 4 vs 1.6 ± 7%), and %Δ BF% (-4.1 ± 7 vs -2.9 ± 3%) (**Figure 3**; all p>0.05). In addition, there was no significant difference between groups in BM, BMI, LMI, and FMI (all p>0.05).

**Table 3:** Body composition results between responder and non-responder groups.

	Responders		Non-responders		P value		
	Pre-CR	Post-CR	Pre-CR	Post-CR	Group	Time	Interaction
Whole body							
BM (kg)	89.6 ± 20	88.4 ± 19^*^	95.5 ± 23	94.0 ± 22	0.13	<0.001	0.34
LBM (kg)	54.3 ± 10	54.7 ± 10^*#^	58.1 ± 11	58.6 ± 12	0.04	0.006	0.77
BFM (kg)	32.5 ± 13	30.7 ± 13^*^	33.7 ± 13	32.4 ± 12^*^	0.43	<0.001	0.37
BL %	61.4 ± 8	62.8 ± 8^*^	61.7 ± 6	62.7 ± 6^*^	0.94	<0.001	0.26
BF %	35 ± 7	34 ± 9^*^	35 ± 6	34 ± 6^*^	0.96	<0.001	0.23
Indexes							
BMI (kg/m^2^)	30.0 ± 6	29.5 ± 6^*^	30.9 ± 6	30.7 ± 6	0.33	<0.001	0.29
LMI (kg/m^2^)	18.1 ± 2	18.2 ± 2^*^	18.9 ± 3	19.1 ± 3	0.07	0.006	0.84
FMI (kg/m^2^)	10.9 ± 4	10.3 ± 4^*^	11.1 ± 4	10.6 ± 3^*^	0.76	<0.001	0.29

Data presented as mean ± SD. CR, cardiac rehabilitation; BM, body mass; LBM, lean body mass; BFM, body fat mass; BL%, body lean percentage; BF%, body fat percentage; BMI, Body mass index; LMI, lean mass index; FMI, fat mass index.

^*^ P<0.05, a significant difference between Pre and Post within the group.

^#^ P<0.05, a significant difference between groups within the Post-CR.

**Figure 3:**
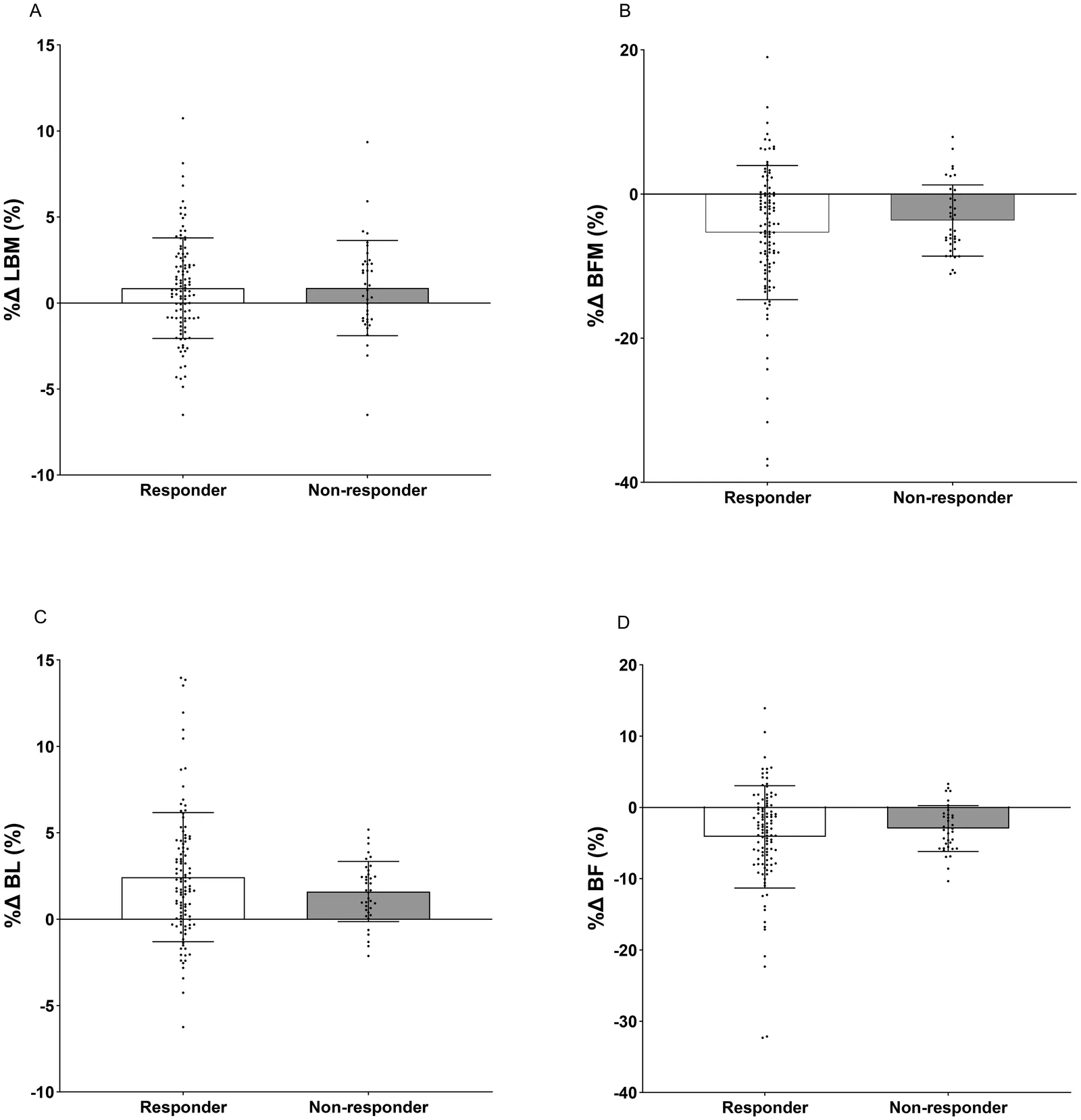
The percent change of body composition variables between responders and non-responders in cohort according to peak relative oxygen consumption. **(A)** Percent change of lean body mass. **(B)** Percent change of body fat mass. **(C)** Percent change of body lean percentage. **(D)** Percent change of body fat percentage.

### Regression models

The univariate regression model showed that no significant baseline body composition or %Δ of body composition, including BM, BLM, BFM, BL%, BF%, BMI, LMI, and FMI, were significantly associated with changes in VO_2_peak (**Table 4**; p>0.05).

**Table 4:** Exploratory unadjusted associations of baseline body composition measures and changes in body composition variables with changes in VO_2_peak.

		Linear regression		
Variable	Coefficient	Standard error	95% confidence interval	P value
1. Baseline				
BM (kg)	-0.09	0.06	-0.19,0.03	0.13
LBM (kg)	-0.0002	0.0001	-0.0004,0.00002	0.07
BFM (kg)	-0.00008	0.00009	-0.0003,0.0001	0.39
BL %	-0.01	0.16	-0.31,0.30	0.97
BF %	-0.39	14.7	−29.4,28.6	0.98
BMI (kg/m^2^)	-0.18	0.20	-0.59,0.22	0.37
LMI (kg/m^2^)	-0.70	0.47	-1.62,0.22	0.13
FMI (kg/m^2^)	-0.09	0.28	-0.64,0.46	0.75
2. Percentage change				
BM (kg)	-0.21	0.37	-0.93,0.51	0.57
BLM (kg)	0.02	0.41	-0.79,0.83	0.97
BFM (kg)	-0.04	0.14	-0.31,0.24	0.78
BL %	0.21	0.35	-0.48,0.91	0.54
BF %	-0.02	0.18	-0.38,0.34	0.91
BMI (kg/m^2^)	-0.21	0.37	-0.93,0.51	0.57
LMI (kg/m^2^)	0.02	0.41	-0.79,0.83	0.96
FMI (kg/m^2^)	-0.04	0.14	-0.31,0.24	0.78

VO_2_, peak oxygen consumption; BM, body mass; LBM, lean body mass; BFM, body fat mass; BL%, body lean percentage; BF%, body fat percentage; BMI, Body mass index; LMI, lean mass index; FMI, fat mass index.

### Absolute VO_2_peak response with cardiac rehabilitation

In the complementary analysis based on absolute VO_2_peak, we found that 69% (n=99) of patients were classified as responders, whereas 31% (n=44) were classified as non-responders. Baseline patient characteristics, CR indications, the interval between CPET assessments, and CPET exercise modality did not differ significantly between groups (all p>0.05; **Supplementary Table 1**). However, non-responders completed significantly fewer CR sessions than responders (P < 0.001; **Supplementary Table 1**).

Among responders, both absolute VO_2_peak and relative VO_2_peak increased significantly by 0.23 ± 0.2 L/min (1.96 ± 0.6 vs 2.2 ± 0.7 L/min) and 2.96 ± 2 mL/kg/min (21.9 ± 5 vs 24.9 ± 6 mL/kg/min), respectively (both p<0.001). For non-responders, both absolute VO_2_peak and relative VO_2_peak decreased significantly by 0.20 ± 0.2 L/min (2.2 ± 0.7 vs 2.0 ± 0.7 L/min) and 1.51 ± 2 mL/kg/min (23.6 ± 7 vs 22.1 ± 7 mL/kg/min), respectively (all p<0.001). In addition, by definition, %Δ absolute VO_2_peaks were significantly different between responders and non-responders (13 ± 8 vs -9 ± 8%; p<0.001). The CPET data based on absolute VO_2_peak response status were generally consistent with those obtained using relative VO_2_peak response status (**Supplementary Table 2**). Compared with non-responders, responders demonstrated significantly greater %Δ exercise time (17 ± 20 vs 4 ± 15), %Δ peak HR (4 ± 15 vs -5 ± 11 beat/min), %Δ AT (10 ± 30 vs -5 ± 22 L/min), and %Δ O2 pulse (9 ± 12 vs -4 ± 10) (**Supplementary Figure 1**; all p<0.05). No significant difference in %Δ rest HR (-3 ± 15 vs -6 ± 15 beat/min) and % ΔHRR (15 ± 235 vs 5 ± 146) between groups (both p>0.05).

Body composition variables based on absolute VO_2_peak response status were also generally consistent with those obtained using relative VO2peak response status. No interaction effect was observed in any body composition variable (all p>0.05; **Supplementary Table 3**), indicating no evidence that the magnitude of body composition changes differed between responders and non-responders. Similarly, the %Δ LBM (0.9 ± 3 vs 0.8 ± 3%), %Δ BFM (-5.4 ± 9 vs -3.7 ± 5%), %Δ BL% (2.4 ± 4 vs 1.8 ± 2%), and %Δ BF% (-4.1 ± 7 vs -3.2 ± 4%) did not differ significantly between responders and non-responders, respectively (**Supplementary Figure 2**; all p>0.05). No significant difference between groups was observed in BM, BMI, LMI, and FMI (all p>0.05).

## Discussion

To our knowledge, this is the first study to examine the association between change in body composition and change in VO_2_peak among patients who completed the CR program. Substantial proportion of patients, specifically 26% based on relative VO_2_peak and 31% based on absolute VO_2_peak, did not experience improvements following the CR program. Our data indicate that body composition variables did not change significantly during the CR program across both responders and non-responders, and the extent of change was comparable between groups. Furthermore, in exploratory unadjusted models, neither baseline body composition variables nor changes in body composition were significantly associated with changes in VO_2_peak among patients who completed the CR program.

In the whole cohort, BMI decreased by 1%, shifting from obese at the Pre-CR to overweight at the post-CR (29). At post-CR, FMI decreased by 5%, which is classified as class I obesity for males and as excess fat for females (30). Our group has previously demonstrated that FMI may be a more accurate measure than BMI in CR patients (29). Prior studies have shown that patients enrolled in CR program demonstrate significant improvement in body composition (21–23, 31). Our results are consistent with a study demonstrating that supervised exercise training significantly improves waist circumference and BF% in non-responder patients with type 2 diabetes (32). Both high BF% and low BL% are associated with an increased risk of cardiovascular events in older people and patients with CAD (18, 33). Therefore, the favorable changes in body composition observed in the whole cohort after the CR program may have potential clinical relevance. However, because the present study did not assess long-term cardiovascular events or mortality, the impact of body composition changes on future clinical outcomes remains uncertain. Future studies should investigate whether improvements in body composition following the CR program are associated with reduced adverse cardiovascular events and mortality, particularly among patients who do not demonstrate an observed increase in VO_2_peak.

Our exploratory unadjusted model suggests that neither baseline body composition variables nor changes in body composition were significantly associated with changes in VO_2_peak after the CR program. Furthermore, the percentage change from pre- to post-CR for all body composition variables did not differ significantly between responders and non-responders despite responders completing significantly more CR sessions. In our previous work identified the number of CR sessions as an independent predictor of VO_2_peak improvement (10). However, the current findings suggest that the association between greater CR sessions and improved VO_2_peak was not accompanied by corresponding differences in body composition changes. Instead, improvements in VO_2_peak may reflect other physiological adaptations that were not assessed in the present study, including enhanced central cardiovascular function, skeletal muscle oxidative capacity, mitochondrial function, microvascular function, and exercise efficiency, rather than measurable changes in body composition. These findings emphasize the multifactorial nature of CRF responses to the CR program and support the need for future mechanistic studies to identify the physiological factors underlying interindividual variability in VO_2_peak improvement (11, 15, 16).

There was an increase in VO_2_peak by 1.6 mL/kg/min in the whole cohort, 2.86 mL/kg/min in responders, and a decrease in VO_2_peak by 2.1 mL/kg/min in non-responders. Clinically, each 1 mL/kg/min increase in VO_2_peak has been associated with a 10% lower risk of all-cause mortality (11). Extrapolating from this, it is conceivable that non-responders may face an elevated risk of all-cause mortality. Therefore, future prospective studies should investigate long-term all-cause mortality among non-responders following the CR program. In addition, our study found that CR sessions were significantly lower among non-responders. Notably, we reported previously that CR sessions are an independent predictor of VO_2_peak change, and each CR session is associated with a 4% increase in VO_2_peak (10). With this, a recent study demonstrated that 8 weeks of exercise training does not increase relative VO_2_peak in patients who underwent the CR program (9), while another study demonstrated that patients achieved the highest relative VO_2_peak after 38 weeks of CR program (34). Altogether, these data suggest that CR sessions are critical to improving VO_2_peak in patients who completed the CR program. Based on these findings, further strategies are needed to maximize CR attendance to improve VO_2_peak in patients undergoing the CR program. This hypothesis warrants further investigation, as it raises pertinent questions about the efficacy of intervention strategies for non-responders and the potential need for alternative or adjunctive therapeutic approaches to mitigate their heightened risk profile.

### Limitation

This study has several limitations that warrant consideration when interpreting the findings and assessing their generalizability. First, this study was a retrospective observational study from a single center; therefore, causation may not be implied. Selection bias may have occurred as only patients with pre- and post-CR CPETs and DEXA were included by design. Second, participants were classified as responders or non-responders using a zero-based threshold for changes in VO_2_peak. This threshold does not account for technical measurement error or normal biological variability inherent to CPET and may therefore overestimate the proportion of patients demonstrating a meaningful physiological response. Because repeated baseline CPET measurements and study-specific estimates of typical error or smallest detectable change were unavailable, an error-based response threshold could not be established. Accordingly, the terms responder and non-responder indicate the presence or absence of an observed increase in VO_2_peak rather than definitive biological response phenotypes. Third, the relatively small and unequal group sizes may have limited the statistical power to detect modest but potentially clinically meaningful associations. Therefore, the absence of statistically significant findings should not be interpreted as definitive evidence that body composition and changes in VO_2_peak are unrelated. In addition, the regression analyses were not adjusted for potential clinical and training-related confounders, including age, sex, baseline VO_2_peak, number of completed CR sessions, and time between CPET assessments. Fourth, detailed clinical information (left ventricular function, comorbidities, smoking status, dietary intake, and cardiovascular medication use) was not consistently available and could not be included in the analyses. Physical activity outside the CR program and the intensity and dose of exercise performed during individual CR sessions were also not recorded. Furthermore, information regarding participation in home-based versus center-based CR during the COVID-19 period and adherence measures beyond the recorded number of completed CR sessions was not consistently available. Accordingly, we could not evaluate whether the findings differed by CR delivery model or fully examine the effects of exercise dose, adherence, and training characteristics on changes in body composition and VO_2_peak. Finally, this study also included predominately males (80%), and the majority of the population in this region (Olmsted County) are White. Therefore, the generalizability of findings is relevant to populations of similar demographics; however, the internal validity of these data remains intact. Therefore, future prospective, multi-center studies with greater patient diversity are needed to understand factors related to the non-response of VO_2_peak after the CR program and to create a new strategy focusing on non-responder patients referred to the CR program.

## Conclusion

In summary, participation in the CR program was associated with favorable changes in body composition in the overall cohort. However, no significant differences in body composition changes were observed between responders and non-responders. Furthermore, neither baseline body composition nor changes in body composition were significantly associated with changes in VO_2_peak during the CR program. Larger prospective studies are warranted to clarify the relationship between body composition and VO_2_peak and to identify the physiological mechanisms underlying interindividual variability in response to the CR program.

## Data Availability

The original contributions presented in the study are included in the article/**Supplementary Material**. Further inquiries can be directed to the corresponding author.
