# Supplementary material for: Relationship between change in body composition and cardiorespiratory fitness during cardiac rehabilitation in patients with cardiovascular disease
Source: Front Endocrinol (Lausanne). 2026 Sep 22;17:1933372. doi: 10.3389/fendo.2026.1933372 (PMC13638497; doi:10.3389/fendo.2026.1933372)
Supplement: Supplementary file 1 [file DataSheet1.docx]

Supplementary Table 1. Baseline characteristics.

|  | Responders  (n=99) | Non-Responders  (n=44) | P-value |
| --- | --- | --- | --- |
| Age (years) | 63±12 | 62±13 | 0.52 |
| Female (%) | 22 (22%) | 6 (14%) | 0.26 |
| Body weight (kg) | 89±19 | 95±24 | 0.09 |
| Height (cm) | 172±9 | 175±8 | 0.06 |
| VO_2_(mL/kg/min) | 21.9±5 | 23.6±7 | 0.12 |
| CR Indication |  |  |  |
| Angina | 7 (7%) | 5 (11%) | 0.51 |
| Acute MI | 14 (14%) | 11 (25%) | 0.15 |
| NSTEMI | 11 (11%) | 2 (5%) | 0.34 |
| STEMI | 6 (6%) | 1 (2%) | 0.44 |
| PCI | 46 (46%) | 21 (48%) | 0.99 |
| Surgery | 13(13%) | 2 (5%) | 0.15 |
| Other | 2 (2%) | 2 (5%) | 0.59 |
| Duration between CPET (weeks) | 15±6 | 14±4 | 0.44 |
| CR sessions | 29±9 | 23±12 | <0.001 |
| CPET modality TM (%) | 92 (93%) | 43 (98%) | 0.44 |

Data presented as mean ± SD or number (%) as appropriate. CR, cardiac rehabilitation; MI, myocardial infarction; NSTEMI, non-ST-elevation myocardial infarction; STEMI, ST-elevation myocardial infarction; PCI, percutaneous coronary intervention; Surgery, coronary artery bypass graft, valves replacement; Other, heart failure, cardiac arrest; CPET, cardiopulmonary exercise test; TM, treadmill.

Supplementary Table 2. Cardiopulmonary exercise test results between responders and non-responders.

|  | Responders | | Non-Responders | | P-value | | |
| --- | --- | --- | --- | --- | --- | --- | --- |
|  | Pre-CR | Post-CR | Pre-CR | Post-CR | Group | Time | Interaction |
| Rest HR (beat/min) | 69±13 | 65±12^*^ | 73±14 | 68±14^*^ | 0.12 | <0.001 | 0.23 |
| Peak HR (beat/min) | 135±23 | 139±19^*#^ | 136±24 | 128±21^*^ | 0.24 | 0.18 | <0.001 |
| HRR (beat/min) | 17±11 | 20±10^*#^ | 14±9 | 16±11 | 0.07 | 0.01 | 0.32 |
| SBP (mm Hg) | 168±28 | 169±26 | 1 69±32 | 161±28 | 0.44 | 0.18 | 0.06 |
| RER | 1.16±0.1 | 1.16±0.1 | 1.14±0.1 | 1.15±0.1 | 0.53 | 0.51 | 0.83 |
| VO_2_ at AT (L/min) | 1.4±0.4 | 1.5±0.5^*^ | 1.6±0.5 | 1.5±0.5 | 0.38 | 0.83 | <0.01 |
| O_2_ pulse (mL/beat) | 14.6±4^‡^ | 15.6±4^*^ | 16.3±4 | 15.8±4^*^ | 0.18 | 0.27 | <0.001 |
| RPE | 18±0.7 | 18±0.7 | 18±0.9 | 18±0.5 | 0.46 | 0.57 | 0.79 |
| Exercise time (min) | 7.6±2 | 8.7±2^*#^ | 7.8±2 | 7.9±2 | 0.27 | <0.001 | <0.001 |

Data presented as mean ± SD. CR, cardiac rehabilitation; HR, heart rate; HRR, heart rate recovery; SBP, systolic blood pressure; RER, respiratory exchange ratio; AT, anaerobic threshold; O_2_ pulse, oxygen pulse; RPE, ratings of perceived exertion.

^*^P<0.05, a significant difference between Pre and Post within the group.

^#^P<0.05, a significant difference between groups within the Post-CR.

^‡^P<0.05, a significant difference between groups within the Pre-CR.

Supplementary Table 3. Body composition results between responder and non-responder groups.

|  | Responders | | Non-Responders | | P value | | |
| --- | --- | --- | --- | --- | --- | --- | --- |
|  | Pre-CR | Post-CR | Pre-CR | Post-CR | Group | Time | Interaction |
| Whole Body |  |  |  |  |  |  |  |
| BM (kg) | 89.2±19 | 97.9±18^*^ | 95.5±24 | 94.3±23 | 0.09 | <0.001 | 0.75 |
| LBM (kg) | 53.9±10^‡^ | 54.4±10^*#^ | 58.3±11 | 58.7±11 | 0.02 | 0.009 | 0.75 |
| BFM (kg) | 32.2±12 | 30.5±12^*^ | 34.0±14 | 32.5±14^*^ | 0.40 | <0.001 | 0.58 |
| BL % | 61±8 | 63±8^*^ | 62±6 | 63±6^*^ | 0.68 | <0.001 | 0.34 |
| BF % | 35±9 | 34±9^*^ | 35±7 | 34±7^*^ | 0.75 | <0.001 | 0.33 |
| Indexes |  |  |  |  |  |  |  |
| BMI (kg/m^2^) | 30±6 | 29±5^*^ | 31±6 | 30±6* | 0.37 | <0.001 | 0.65 |
| LMI (kg/m^2^) | 18.0±2 | 18.2±2^*^ | 18.9±3 | 19.0±3 | 0.07 | 0.007 | 0.68 |
| FMI (kg/m^2^) | 11 ±4 | 10±4^*^ | 11±4 | 10±4^*^ | 0.90 | <0.001 | 0.46 |

Data presented as mean ± SD. CR, cardiac rehabilitation; BM, body mass; LBM, lean body mass; BFM, body fat mass; BL%, body lean percentage; BF%, body fat percentage; BMI, Body mass index; LMI, lean mass index; FMI, fat mass index.

^*^P<0.05, a significant difference between Pre and Post within the group.

^#^P<0.05, a significant difference between groups within the Post-CR.

^‡^P<0.05, a significant difference between groups within the Pre-CR.

**Supplementary Figure 1**

**Figure 1.** The percent change of cardiopulmonary exercise test variables between responders and non-responders in cohort according to peak absolute oxygen consumption. (A) Percent change of exercise time (B) Percentage change of peak heart rate. (C) Percent change of anaerobic threshold. (D) Percent change of oxygen pulse.

****P<0.0001, ***P<0.001, **P<0.01

**Supplementary Figure 2**

**Figure 2.** The percent change of body composition variables between responders and non-responders in cohort according to peak absolute oxygen consumption. (A) Percent change of lean body mass. (B) Percent change of body fat mass. (C) Percent change of body lean percentage. (D) Percent change of body fat percentage.
